# Raspberry alleviates obesity-induced inflammation and insulin resistance in skeletal muscle through activation of AMP-activated protein kinase (AMPK) α1

**DOI:** 10.1038/s41387-018-0049-6

**Published:** 2018-07-02

**Authors:** Liang Zhao, Tiande Zou, Noe Alberto Gomez, Bo Wang, Mei-Jun Zhu, Min Du

**Affiliations:** 1 Department of Animal Sciences, Nutrigenomics and Growth Biology laboratory, Washington State University, Pullman, WA 99164 USA; 2 Jiangxi Province Key Laboratory of Animal Nutrition, College of Animal Science and Technology, Jiangxi Agricultural University, Nanchang, Jiangxi 330045 China; 3 School of Food Sciences, Washington State University, Pullman, WA 99164 USA; 4 Beijing Advanced Innovation Center for Food Nutrition and Human Health, College of Food Science & Nutritional Engineering, China Agricultural University, Beijing, 100194 China

## Abstract

**OBJECTIVE:**

Through dynamic means, etiological factors, including chronic inflammation and insulin resistance have the potential to perpetuate metabolic incidences such as type 2 diabetes and obesity. Abatement of such syndromes can be achieved by complex mechanisms initiated through bioactive compounds such as polyphenols derived from fruits. Using a whole-fruit approach, the effects of dietary red raspberry, which is rich in polyphenols, on inflammatory responses and insulin resistance in the skeletal muscles of *Mus musculus* were studied along with the potential role of AMP-activated protein kinase (AMPK) to act as a key mediator.

**SUBJECTS:**

Wild-type (WT) mice and mice deficient in the catalytic subunit (α1) of AMPK (AMPKα1^−^^/−^) were fed with a high-fat diet (HFD) or HFD supplemented with raspberry (5% dry weight) for 10 weeks. Factors involved in inflammatory responses, insulin signaling transduction, and mitochondrial biogenesis were evaluated.

**RESULTS:**

Dietary raspberry reduced ectopic lipid storage, alleviated inflammation responses, improved whole-body insulin sensitivity, and promoted mitochondrial biogenesis in the skeletal muscle of WT mice, but not AMPKα1^−/−^ mice.

**CONCLUSIONS:**

AMPKα1 is an important mediator for the beneficial effects of raspberry through alleviating inflammatory responses and sensitizing insulin signaling in skeletal muscle of HFD-fed mice.

## INTRODUCTION

Red raspberry is widely recognized for its high levels of vitamin C and bioactive polyphenols, including ellagitannins and anthocyanins, which have strong antioxidant capacities^1^. Several animal studies have shown that supplementation of raspberry extracts exhibited beneficial effects for the prevention of obesity, inflammation and other metabolic diseases^2, 3^. However, the impacts of dietary raspberry fruit on skeletal muscle insulin resistance and the underlying mechanisms remain largely unexplored.

Obesity induces ectopic lipid accumulation and desensitizes insulin signaling in skeletal muscle, thus resulting in systematic insulin resistance and type 2 diabetes^4^. AMP-activated protein kinase (AMPK) is a key sensor of energy status in skeletal muscle through the control of glucose and fatty acid metabolism^5^. The structure of AMPK has been described as a heterotrimeric complex comprised of the catalytic α-subunit and the regulatory β- and γ- subunits^6^. Activation of AMPK prevents obesity and associated metabolic diseases through the promotion of glucose utilization, fatty acid oxidation, and mitochondrial biogenesis in skeletal muscle^6^. Dietary polyphenols, such as resveratrol, are strong activators of AMPK, which can then promote the browning of white adipose and subsequently alleviate obesity^7^. Due to the high levels of polyphenols found in the red raspberry, it is postulated that AMPK plays an essential role in mediating the beneficial effects of red raspberry on metabolic health.

The catalytic subunit of AMPK has 2 isoforms (α1 and α2). Although there is a compensatory mechanism between these two isoforms, their expression shows tissue-specific patterns^8, 9^ with differential metabolic functions^10, 11^. The isoform α2 of AMPK is indispensable for increased glucose uptake by skeletal muscle induced by 5-aminoimidazole-4-carboxamide ribonucleotide (AICAR) and hypoxia^5, 12, 13^. Meanwhile, the AMPKα1 isoform can be activated during skeletal muscle contraction^14^ and at low caffeine concentrations^10^. Indeed, AMPKα1 also plays an essential role in myogenin expression and myogenesis^15^. Previous studies in our lab have shown the dominant expression of AMPKα1 in satellite cells, which when deleted, impeded muscle regeneration after injury^15^. Deletion of AMPKα1 in macrophages during the transition from a proinflammatory (M1) to an anti-inflammatory (M2) phenotype impairs the resolution of inflammation and muscle regeneration after injury^16^. Altogether, these studies suggested that AMPKα1 could mediate the alleviation of insulin resistance and metabolic syndromes in skeletal muscle of obese mice consuming raspberry. Thus, we explored the influence of red raspberry on insulin sensitivity and inflammatory responses in skeletal muscles, along with the potential role of AMPKα1 to act as a key mediator.

## MATERIALS AND METHODS

### Animal and experimental design

R26^Cre^/AMPKα1^fl/fl^ mice were generated through the cross-breeding of AMPKα1^fl/fl^ mice (Stock No: 014141, Jackson Lab, Bar Harbor, Maine) with tamoxifen-inducible R26-Cre mice (Stock No: 004847, Jackson Lab, Bar Harbor, Maine) at Washington State University. To induce the AMPKα1 knockout (AMPKα1^−/−^), 2-month-old male R26^Cre^/AMPKα1^fl/fl^ mice were intraperitoneally injected with tamoxifen (75 mg/kg body weight) for 4 continuous days^17^. AMPKα1^fl/fl^ mice treated with tamoxifen were used as controls (Wild-type, WT). To minimize possible confounding changes, dietary treatments started 3 days after the last tamoxifen injection^15^. All experimental procedures of animal use were performed according to the guidelines of National Institutes of Health and approved by the Animal Use and Care Committee of Washington State University (Permit No. 04719).

Twelve wild-type and AMPKα1^−/−^ mice, respectively, were randomly separated into two sub-groups and fed either a high-fat diet (HFD; 60% energy from fat, D12492; Research Diets, New Brunswick, NJ, USA) or a HFD diet supplemented with freeze-dried raspberry (5% of dry feed weight, red raspberry powder). The concentration of the raspberry supplementation was determined by preliminary studies in our lab^18^. Raspberry powder was prepared as previously described, which contains polyphenols at ~11 g gallic acid equivalent (GAE)/kg of dry weight, 4.24 ± 0.12% protein, 1.91 ± 0.03% fat, 0.81 ± 0.02% ash, 16.14 ± 0.45% moisture, and the remaining to be mainly carbohydrates^19^. Mice were housed in a temperature-controlled environment (23 ± 2 °C, alternating 12-h light/dark cycle) with *ad libitum* access to food and water. Feed intake and body weights were monitored weekly until the mice were killed 10 weeks later. Samples of blood, the *Gastrocnemius* muscle (GA), and the *Tibialis anterior* muscle (TA) were rapidly isolated. TA were fixed in 4% paraformaldehyde for sectioning and staining, and GA were rapidly frozen in liquid nitrogen and stored at −80 °C until further analyses.

### Histochemical analyses

Paraffin-embedded TA muscle sections (5-μm thick) were rehydrated through a series of incubations in xylene and ethanol solutions, and then used for Masson trichrome staining^20^. At least four fields per section and four sections per sample were randomly selected for quantification of fat area and collagen area using the Image J 1.46r software (National Institutes of Health). The average data per biological sample were used for calculations.

### Total triacylglycerol analyses

As previously described, total triacylglycerol determination was performed using the Folch method^20, 21^. The frozen GA muscle was powdered under liquid nitrogen and a 30 mg sample was weighed. After adding 0.75 ml of chloroform-methanol 2:1 (v/v), the samples were left at 4 °C for 48 h. Then, 187.5 µl 0.9% NaCl was added and the mixture was kept at room temperature overnight and then centrifuged at 10,000 × *g* for 5 min at 4 °C. The lower phase (20 µl) was transferred into a fresh tube and evaporated until dry for 1 h under the hood. Total triacylglycerols were measured using a kit from Sigma following the manufacturer’s instructions (cat. no. TR0100). The results were displayed by dividing the total triacylglycerol content by the initial muscle powder weight.

### Quantitative real-time PCR (qRT-PCR) analyses

Total RNA was isolated using TRIzol reagent (Sigma, Saint Louis, MO, USA), followed by reverse-transcription to cDNA using the iScriptTM cDNA Synthesis Kit (Bio-Rad, Hercules, CA, USA). The mRNA levels were measured by qRT-PCR carried out by the CFX RT-PCR detection system (Bio-Rad). After normalization to *18s* rRNA content, relative mRNA expression was determined using the method of 2^-ΔΔCt^^22^. Table 1 shows the primer sequences.

**Table 1:** Primer sequences used for real-time quantitative PCR

Gene	Forward (5′–3′)	Reverse (5′–3′)	Size (bp)	Access No.
*18s*	TTGTACACACCGCCCGTCGC	CTTCTCAGCGCTCCGCCAGG	102	NR_003278.3
*Glut4*	CTCTCAGGCATCAATGCTGTTTTCTA	CGAGACCAACGTGAAGACCGTATT	123	NM_001359114.1
*Il1β*	TCGCTCAGGGTCACAAGAAA	CATCAGAGGCAAGGAGGAAAAC	73	XM_006498795.3
*Il6*	GAGGATACCACTCCCAACAGACC	AAGTGCATCATCGTTGTTCATACA	141	NM_001314054.1
*Il18*	ATGCTTTCTGGACTCCTGCCTGCT	GGCGGCTTTCTTTGTCCTGATGCT	89	XM_006510028.3
*Tnfα*	TGGGACAGTGACCTGGACTGT	TTCGGAAAGCCCATTTGAGT	67	NM_001278601.1
*Cycs*	CCAAATCTCCACGGTCTGTT	GTCTGCCCTTTCTCCCTTCT	192	NM_007808.4
*Pgc1α*	CCCTGCCATTGTTAAGACC	TGCTGCTGTTCCTGTTTTC	161	XM_006503779.1
*Nrf1*	GCACCTTTGGAGAATGTGGT	CTGAGCCTGGGTCATTTTGT	165	NM_001164226.1
*Cpt1*	GTCGCTTCTTCAAGGTCTGG	AAGAAAGCAGCACGTTCGAT	232	NM_009948.2
*Tfam*	CCAAAAAGACCYCGTTCAGC	CTTCAGCCATCTGCTCTTTCC	211	NM_009360.4

### Immunoblotting analyses

Immunoblotting analyses were performed as previously described using the Odyssey Infrared Image System (LI-COR Biosciences, Lincoln, NE, USA)^15^. Band densities of target proteins were normalized to β-tubulin content. The following antibodies were purchased from Cell Signaling (Danver, MA, USA): AMPKα (no.2532), phospho-AMPKα at Thr172 (no. 2535), protein kinase B (AKT, no.9272), phospho-AKT at Ser473 (no. 9271), protein kinase C (PKCθ, no.13643), phospho-PKCθ at Thr538 (no.9377), nuclear factor κB (NFκB) subunit p65 (no.8242), phospho-p65 at Ser536 (no.3033), c-Jun N-terminal kinases (JNK, no. 9252), phospho-JNK at Thr183/Tyr185 (no.9251) and cytochrome C (cyt C, no. 4280). IRDye 800CW goat anti-rabbit (no. 926-32211) and IRDye 680 goat anti-mouse (no. 926-68070) secondary antibodies were purchased from LI-COR Biosciences (Lincoln, NE, USA). For use, primary antibodies were diluted 1: 1000 using 1× TBST buffer (137 mM Sodium Chloride, 20 mM Tris, 0.1% Tween-20, pH 7.6) with 5% BSA (Bovine Serum Albumin) and secondary antibodies were diluted 1: 10,000 using TBST buffer.

### Statistical analyses

Within each genotype, the data were analyzed using unpaired two-tailed Student’s *t* test using SAS 9.0 (SAS Institute Inc., Cary, NC, USA). All the data were found normally distributed. Results are expressed as mean ± s.d. A significant difference was considered as *P* < 0.05.

## RESULTS

### Raspberry supplementation activated AMPKα1

**Fig. 1:**
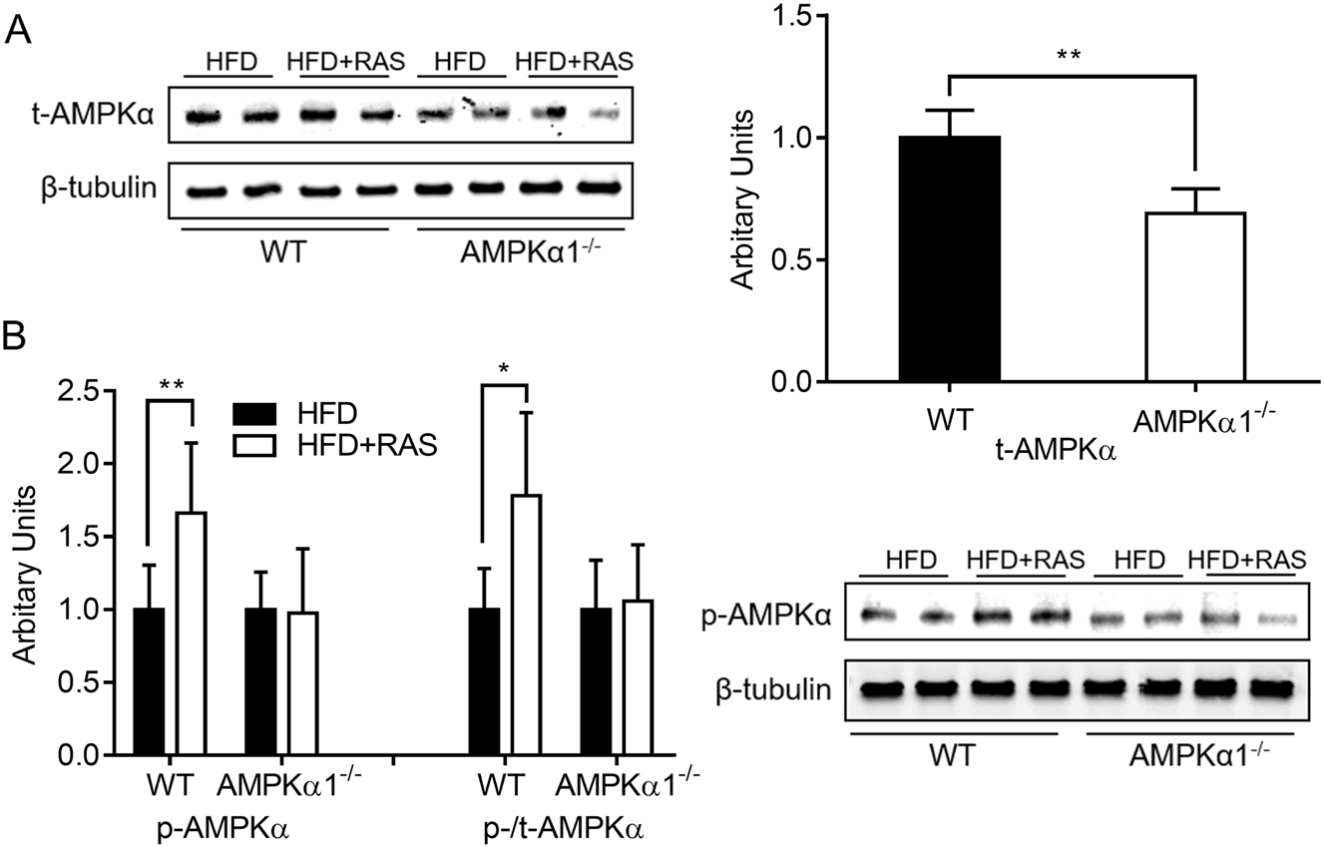
Contents of total AMPKα (**a**) and phospho-AMPKα (**b**) in the *Gastrocnemius* muscle of wild-type (WT) and AMPKα1 knockout mice with/without raspberry supplementation. (**P < 0.01, *P < 0.05, HFD + RAS vs. HFD only for each genotype as determined by unpaired two-tailed Student’s *t* test; mean ± s.d.; *n* = 6). t total content, p phosphorylated form

The content of total AMPKα in skeletal muscles was lower in AMPKα1^−/−^ mice (Fig. 1a), which is consistent with successful AMPKα1 knockout induced by tamoxifen. Raspberry supplementation increased the level of p-AMPKα and the ratio of p-/t-AMPK in WT mice, while no difference was found in AMPKα1^−/−^ mice with/without raspberry (Fig. 1b). The lack of difference in AMPK phosphorylation and ratio of p-/t-AMPK in the absence of AMPKα1 suggests that raspberry supplementation did not activate AMPKα2.

### Raspberry supplementation reduced lipid accumulation in skeletal muscles in an AMPKα1-dependent manner

**Fig. 2:**
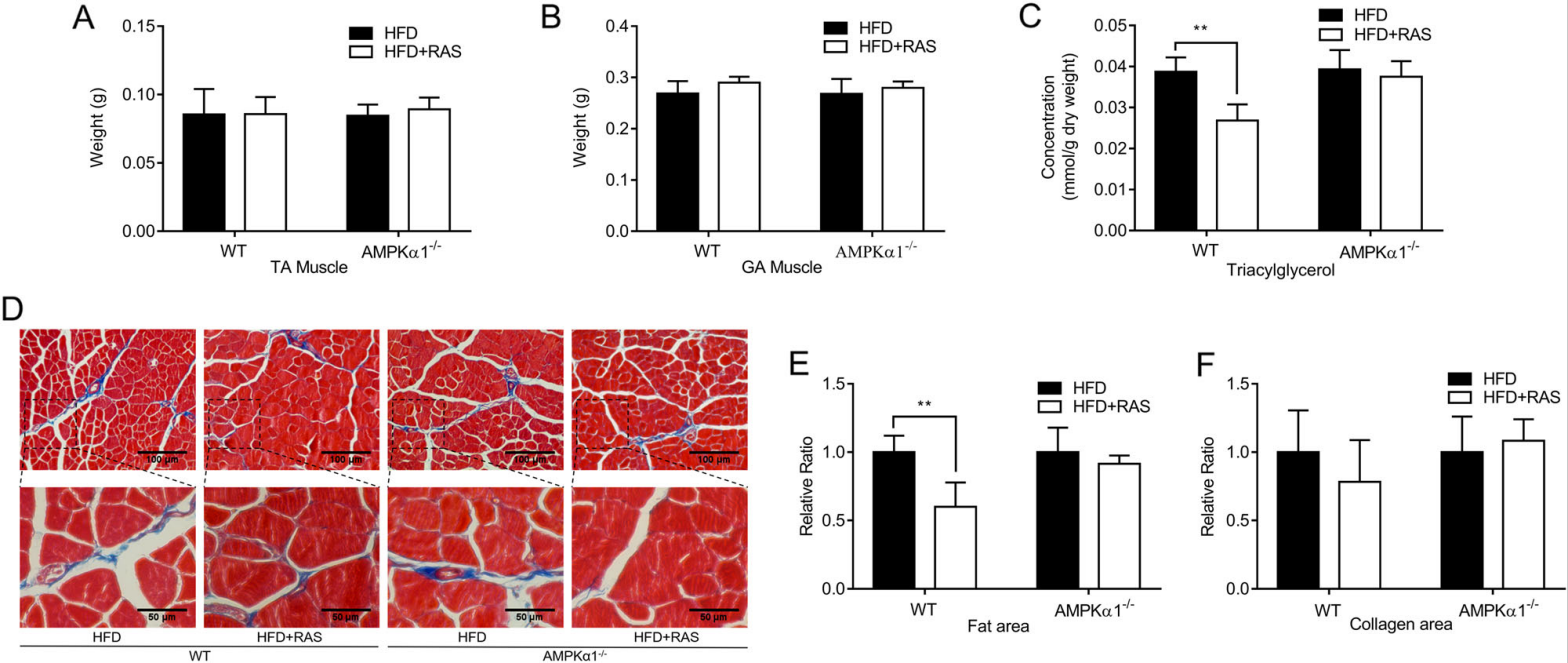
Muscle weight and ectopic lipid accumulation in the skeletal muscle of wild-type (WT) and AMPKα1 knockout mice with/without raspberry supplementation. **a** *Tibialis anterior* muscle weight. **b** *Gastrocnemius* muscle weight. **c** Total triacylglycerol content in *Gastrocnemius* muscle. **d** Masson trichrome staining of *Tibialis anterior* (top: ×200 magnification; bottom: ×400 magnification) and relative area of fat (**e**) and collagen (**f**). (**P < 0.01, *P < 0.05, HFD + RAS versus HFD only for each genotype as determined by unpaired two-tailed Student’s *t* test; mean ± s.d.; *n* = 6)

As described previously in our lab, there was no significant difference of average weekly food intake between groups (*p* > 0.05) and dietary raspberry reduced the body weight of wide type mice but not that of the AMPKα1^−/−^ mice (*p* < 0.01)^23^. The TA and GA muscle weights were not altered through raspberry supplementation, nor by AMPK α1 deficiency (Fig. 2a, b).

Intramuscular lipid accumulation contributes to obesity-induced insulin resistance by activating stress-responsive serine kinases and then impeding the activity of downstream insulin signaling molecules such as AKT^24, 25^. The triacylglycerol content in the GA muscle was elevated due to the HFD, but partially prevented by dietary raspberry in WT mice. For the AMPKα1 KO mice, no difference was found between HFD and HFD + RAS groups (Fig. 2c), supporting the mediatory role of AMPK α1.

Masson trichrome staining shows the areas of muscle cells in red, collagen in blue, and adipocytes as colorless. More intramuscular adipocytes in TA muscle were observed in the HFD group compared to the HFD + RAS group of WT mice as shown in Fig. 2d. The areas of fat (Fig. 2e) and collagen (Fig. 2f) in muscle sections were quantified. Fat area was much smaller (*P* < 0.01) in the HFD + RAS group compared to the HFD group of WT mice, consistent with the lower levels of triacylglycerols in the HFD + RAS group of WT mice as shown in Fig. 2c. Raspberry supplementation also decreased the presence of connective tissues in WT mice. A tendency for a decrease in collagen area was seen in the HFD + RAS group of WT mice (*P* < 0.10). For AMPKα1^−/−^ mice, no significant difference was exhibited for either fat and collagen areas.

These data suggest that raspberry supplementation reduced lipid accumulation in skeletal muscle of mice challenged with a HFD diet, a process mediated by AMPK α1.

### Raspberry supplementation decreased the inflammatory response in an AMPKα1-dependent manner

**Fig. 3:**
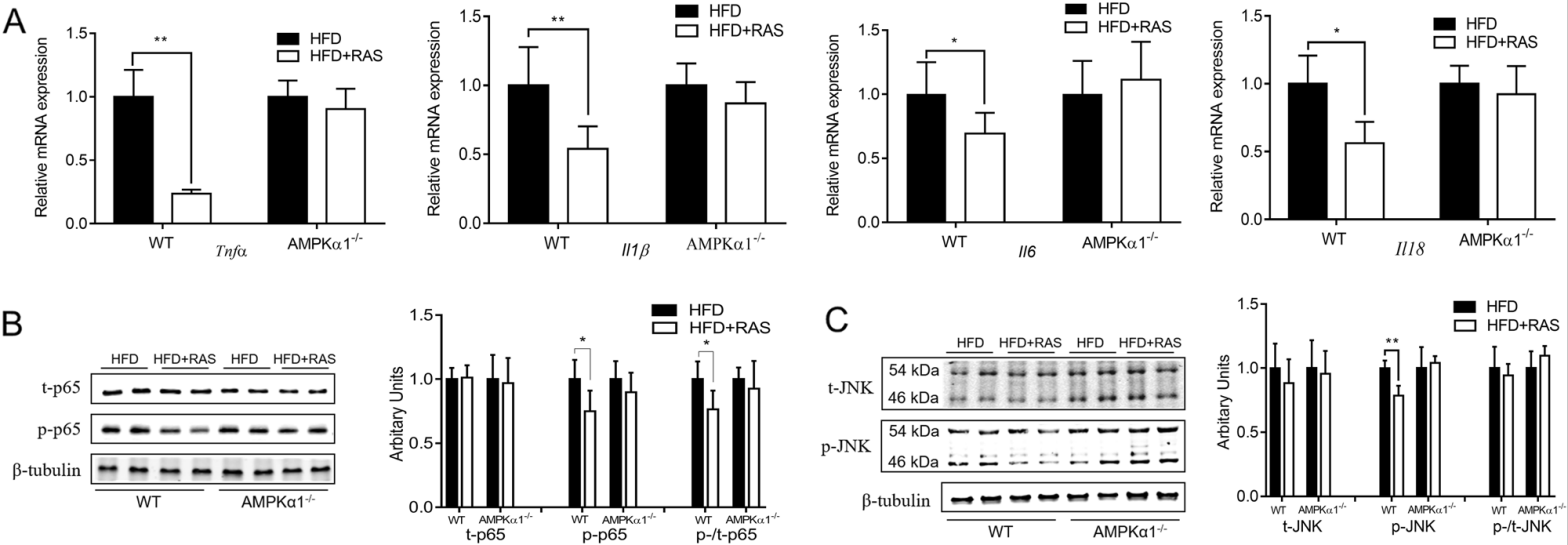
Inflammatory response in the *Gastrocnemius* muscle of wild-type (WT) and AMPKα1 knockout mice with/without raspberry supplementation. **a** Quantitative PCR analysis of *Tnfα*, *Il1β*, *Il6* and *Il18* expression. **b** Western blots of t-p65 and p-p65 and their relative contents. **c** Western blots of t-JNK and p-JNK and their relative contents. (**P < 0.01, *P < 0.05, HFD + RAS vs. HFD only for each genotype as determined by unpaired two-tailed Student’s *t* test; mean ± s.d.; *n* = 6). t total content, p phosphorylated form

Ectopic lipid accumulation in peripheral tissues frequently leads to chronic inflammation. Raspberry intake attenuated HFD-stimulated expression of *Tnfα*, *Il1β*, *Il6*, and *Il18* in WT mice (Fig. 3a). However, this beneficial role of raspberry supplementation was not present in AMPKα1^−/−^ mice.

Inflammatory responses are mediated by the activation of NF-κB (nuclear factor kappa B) and JNK/MAPK pathways^26–28^. Protein p65 is a key component of the NF-κB pathway with obesity up-regulating its phosphorylation^20^. Although the total contents of p65 did not change, a much lower phosphorylation level of p65 and a low phospho to total ratio of p65 (p-p65/t-p65) were detected in the HFD + RAS group of WT mice (Fig. 3b). In AMPKα1^−/−^ mice, raspberry supplementation did not reduce the phosphorylation level of p65. In addition, raspberry supplementation also decreased the phosphorylation level of JNK in WT mice (Fig. 3c). Although the total level of JNK and the ratio of p-/t-JNK showed a decreasing tendency, changes were not significant. However, these benefits disappeared in AMPKα1^−/−^ mice, showing the mediatory role of AMPKα1.

### Raspberry improved insulin sensitivity in an AMPKα1-dependent manner

Previous studies in our laboratory have reported that raspberry supplementation increased glucose tolerance, and decreased lipids and insulin levels in the serum of WT mice but not in AMPKα1^−/−^ mice, which reflected improved insulin sensitivity by raspberry supplementation through regulating AMPKα1^23^.

**Fig. 4:**
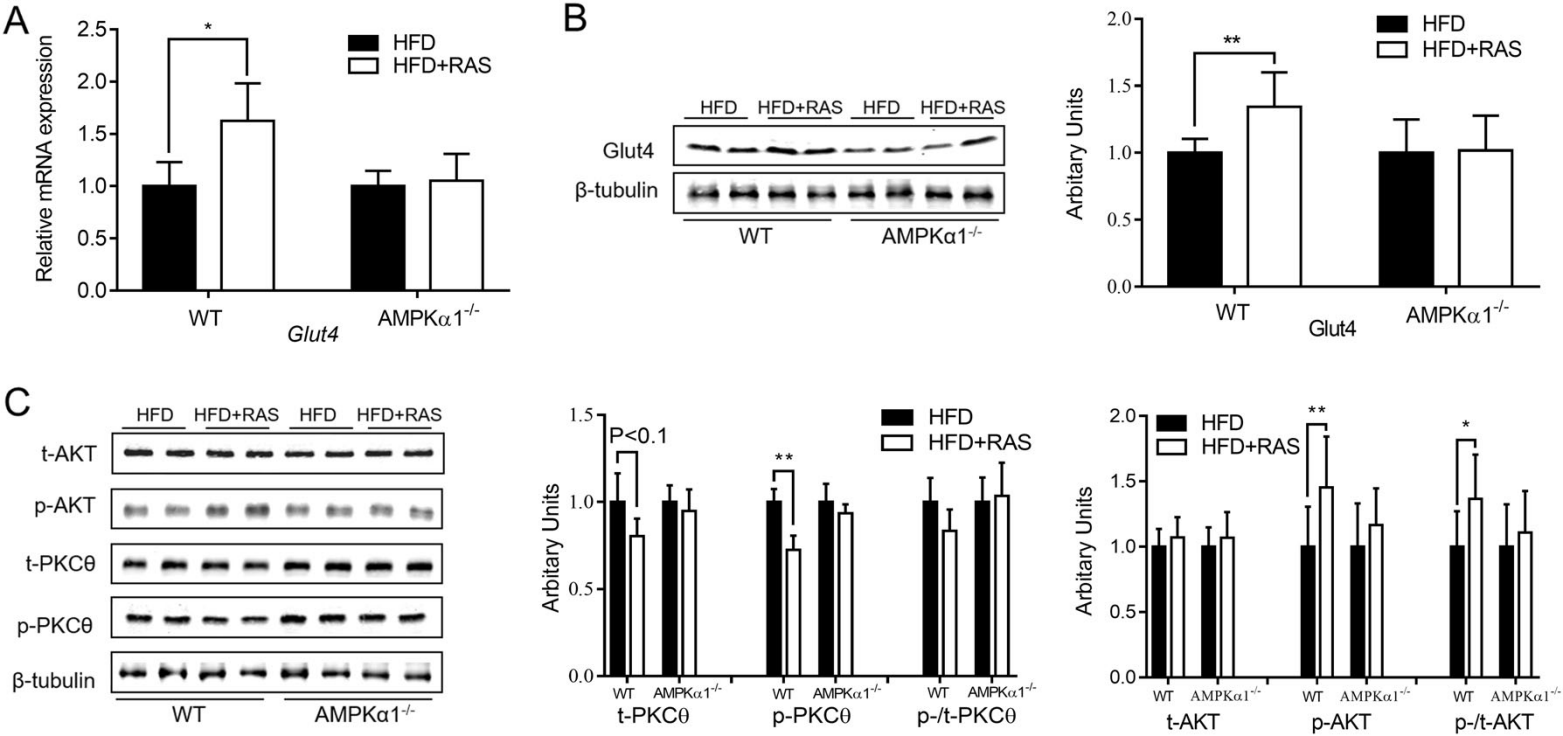
Expression of Glucose transport 4 (GLUT4) and relative contents of insulin signaling proteins in the *Gastrocnemius* muscle of wild-type (WT) and AMPKα1 knockout mice with/without raspberry supplementation. **a** Quantitative PCR analysis of *Glut4* expression. **b** Western blots of Glut4 and their relative contents. **c** Western blots of insulin signaling proteins (t-AKT, p-AKT, t-PKCθ and p-PKCθ) and their relative contents. (**P < 0.01, *P < 0.05, HFD + RAS versus HFD only for each genotype as determined by unpaired two-tailed Student’s *t* test; mean ± s.d.; *n* = 6), t total content, p phosphorylated form

Glucose transporter 4 (GLUT4) is indispensable for whole-body glucose homeostasis and its deficiency leads to insulin resistance and ectopic lipid accumulation^29, 30^. Consistently raspberry supplementations increased *Glut4* mRNA and protein contents in WT mice but not in AMPKα1^−/−^ mice (Fig. 4a, b).

Because increased lipid accumulation and inflammation are correlated with insulin resistance, insulin signaling pathways were further analyzed. In WT mice, the contents of PKCθ and its phosphorylation were down-regulated by 19.5% (*p* < 0.1) and 27.5% (*p* < 0.01) in raspberry supplemented group, respectively. In the absence of AMPKα1, however, these differences disappeared (Fig. 4c).

Although the total level of AKT was not different, its phosphorylation was higher (*P* < 0.01) in RAS supplemented WT mice when compared to those fed only HFD (Fig. 4c). Consequently, the HFD + RAS group of WT mice had a significantly higher p-/t-AKT ratio (*P* < 0.05). Ablation of AMPKα1 abolished these changes induced by raspberry supplementation. Therefore, AMPKα1 is required for the beneficial effects of raspberry on insulin signaling in skeletal muscle of mice under the challenge of HFD.

### Raspberry promoted mitochondrial biogenesis in an AMPKα1-dependent manner

**Fig. 5:**
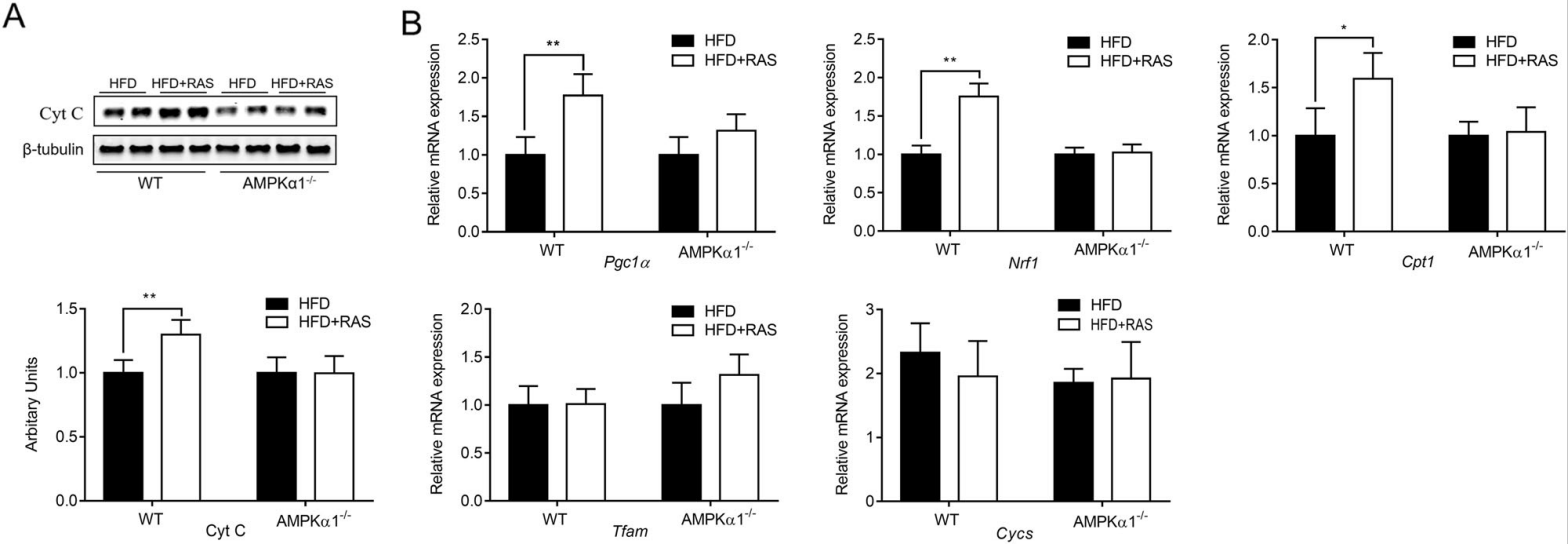
Mitochondria biogenesis in the *Gastrocnemius* muscle of wild-type (WT) and AMPKα1 knockout mice with/without raspberry supplementation. **a** Western blots cytochrome C (Cyt C) and its relative contents. **b** Quantitative PCR analysis of *Pgc1α*, *Nrf1*, *Cpt1*, *Tfam* and *Cycs*. (**P < 0.01, *P < 0.05, HFD + RAS versus HFD only for each genotype as determined by unpaired two-tailed Student’s *t* test; mean ± s.d.; *n* = 6)

The mitochondria play an indispensable role in cellular energy metabolism while its dysfunction in skeletal muscle is associated with decreased insulin sensitivity and the development of type 2 diabetes^31^. Raspberry supplementation increased the protein level of cytochrome C (Cyt C) in skeletal muscle (*p* < 0.01), suggesting increased contents of mitochondria (Fig. 5a). Meanwhile, the mRNA expression levels for *Pgc1α*, *Nrf1*, and *Cpt1* were up-regulated in the HFD + RAS group of WT mice (Fig. 5b). However, in AMPK α1^−/−^ mice, no such differences were observed. The mRNA expression of *Cycs* and *Tfam* did not differ between WT and AMPK α1^−/−^ groups. In summary, increased mitochondrial biogenesis could be responsible for the reduced lipid accumulation elicited by raspberry supplementation in WT mice challenged with HFD in an AMPKα1-dependent manner.

## DISCUSSION

Obesity and associated chronic inflammations induce a state of insulin resistance in adipose tissue, skeletal muscles, and the liver, which is indispensable for the development of type 2 diabetes^32^. Numerous pharmaceutical approaches aimed at preventing obesity and inflammation have shown positive results, but with various side effects and risks^33^. Nutritional interventions have the advantage of being natural and safe, providing a more suitable alternative for long-term therapy. Raspberries contain high amounts of polyphenols and other bioactive compounds and have been shown to have beneficial effects in treating obesity and metabolic diseases^3, 34^. However, the effects of raspberry in insulin resistance of skeletal muscle and the mediatory role of AMPK have not been examined.

Obesity induces ectopic lipid storage and inflammatory response, accompanied by the secretion of proinflammatory cytokines such as TNFα, IL1β and IL6^31^. The bioactive polyphenols in red raspberry occur primarily as ellagitannins and anthocyanins, which have anti-inflammatory effects^1, 35^. In the current study, raspberry supplementation promoted insulin signaling, reduced lipid accumulation, and alleviated the inflammatory response in skeletal muscle. These benefits disappeared in AMPKα1 knockout mice, which showed the indispensable role of AMPKα1 in mediating the beneficial effects of dietary raspberry. Increased mitochondrial biogenesis in WT mice due to raspberry consumption could be a causative reason for these beneficial effects. Following AMPKα1 knockout, raspberry-stimulated mitochondrial biogenesis disappeared, supporting the mediatory role of AMPKα1.

AMPK is a promising drug target for preventing and treating obesity and associated metabolic disease^36^. Increasing the activity of AMPK in skeletal muscles is associated with enhanced mitochondrial biogenesis and lipid oxidation^7^. The two catalytic α isoforms (α1 and α2) of AMPK have different tissue expression patterns. AMPKα1 is widely expressed in all tissues while predominately in brain and adipose tissues, whereas both α1 and α2 isoforms are expressed in skeletal muscles and the heart^37^. Their difference in subcellular localization and substrate specificity also suggest their differential roles in the regulation of metabolic processes^8, 9^. AMPK is normally activated in response to an energy-depleting state^17^. Due to allosteric activation by AMP and covalent activation by upstream kinases, AMPKα2 activation is more dependent on AMP and energy depletion than the α1 isoform^8, 38^. Isoforms of AMPK are activated according to the intensity of exercise: low-intensity exercise preferentially activates the α1 isoform while moderate intensity exercise preferentially activates the α2 isoform^11^. In obesity and insulin resistance models, endurance training (treadmill running) increased the activity of AMPKα1 but not the α2 isoform^39, 40^. The mediating role of caffeine (1,3,7-trimethylxanthine) on skeletal muscle metabolism is also achieved through AMPK; low concentrations (1 mM) of caffeine predominantly activate AMPKα1 via an energy-independent manner while AMPKα2 was activated at high concentrations (3 mM) of caffeine, depending on energy depletion^10^. The polyphenols in red raspberries, such as anthocyanins, activate AMPKα1^41^, consistent with our observation in this study that dietary raspberry did not significantly activate AMPKα2 in the skeletal muscle of obese mice.

In conclusion, we found that raspberry supplementation reduced lipid accumulation, alleviated the inflammatory response, improved insulin sensitivity, and promoted mitochondrial biogenesis in the skeletal muscle of HFD-fed mice. These beneficial effects depended on the indispensable mediator: AMPKα1. Further studies should focus on the signaling mechanisms of raspberry with regards to the isoform-specific activation of AMPK, which could provide new insights for the development of dietary treatments for reducing obesity and diabetes.
